# Impact of Early Physiotherapy Rehabilitation for Pleural Empyema in a Geriatric Patient

**DOI:** 10.7759/cureus.28158

**Published:** 2022-08-19

**Authors:** Aditi Joshi, Moli Jain, Vishnu Vardhan

**Affiliations:** 1 Physiotherapy, Ravi Nair Physiotherapy College, Wardha, IND; 2 Physiotherapy, Datta Meghe Institute of Medical Sciences, Wardha, IND; 3 Department of Cardiorespiratory Physiotherapy, Ravi Nair Physiotherapy College, Datta Meghe Institute of Medical Sciences, Wardha, IND

**Keywords:** physiotherapy management, intercostal drainage, thoracoscopy, pleural effusion, empyema

## Abstract

Empyema is the accumulation of pus in the pleural cavity which can be linked to lung abscesses, trauma, septicemia, or spinal osteomyelitis. It is usually caused by a lung infection that extends to the pleural space and causes pus to accumulate. Here, we present the case of a 70-year-old male who complained of dry cough for 15 days, breathlessness on walking for 20 days, right-sided chest and upper back pain, and high-grade fever for 15 days. On investigation, pleural empyema was diagnosed. He underwent a thoracoscopy to drain the fluid and an intercostal drainage tube was inserted. Along with medical management, physiotherapy was also required to help the patient to perform his daily activities with ease. A physiotherapy protocol was developed for the patient to improve his condition.

## Introduction

Pleural empyema is a serious infection-related complication that rarely resolves without appropriate medical therapy and drainage procedures. Empyema is the accumulation of pus in the pleural cavity which can be linked to lung abscesses, trauma, septicemia, or spinal osteomyelitis. It is usually caused by a lung infection that extends to the pleural space and causes pus accumulation [1]. Clinical signs include persistent fever and pleural involvement. Tuberculosis is also one of the causes of pleural empyema usually in the elderly as immunity decreases with age and other degenerative processes in the body begin to occur [2]. On the other hand, empyema can also be a secondary cause of pleural effusion. Pleural effusion is an excessive accumulation of fluid in the pleural cavity. It can be secondary to pneumonia, tuberculosis, malignancy of the lungs, lung abscess, lymph node blockage, and many more diseases. Usually, patients have dyspnea, cyanosis if the effusion is large, pain, lethargy, and restricted thoracic movements [3]. The pleural layers come together and may become adherent leading to the organization of fibrin due to the presence of plasma proteins in the fluid. The presence of fibrous tissue leads to restricted lung mobility, eventually causing alteration in the breathing pattern of the patient [4]. Patients require multidisciplinary treatment. Along with medications, physiotherapy also plays a crucial role in the treatment protocol for pleural effusion as well as in pleural empyema. The aim of physiotherapy is to avoid the formation of disabling adhesions between two pleura layers, regain full lung expansion, improve the ventilation of the lungs, improve the exercise tolerance capacity, and maintain joint mobility [3,4]. In this case, the patient was diagnosed with pleural empyema post-pleural effusion a few months ago and was undergoing treatment and rehabilitation for improvement in his condition. He was referred to the physiotherapy department where a proper well-planned treatment protocol was developed for the patient.

## Case presentation

**Figure 1:**
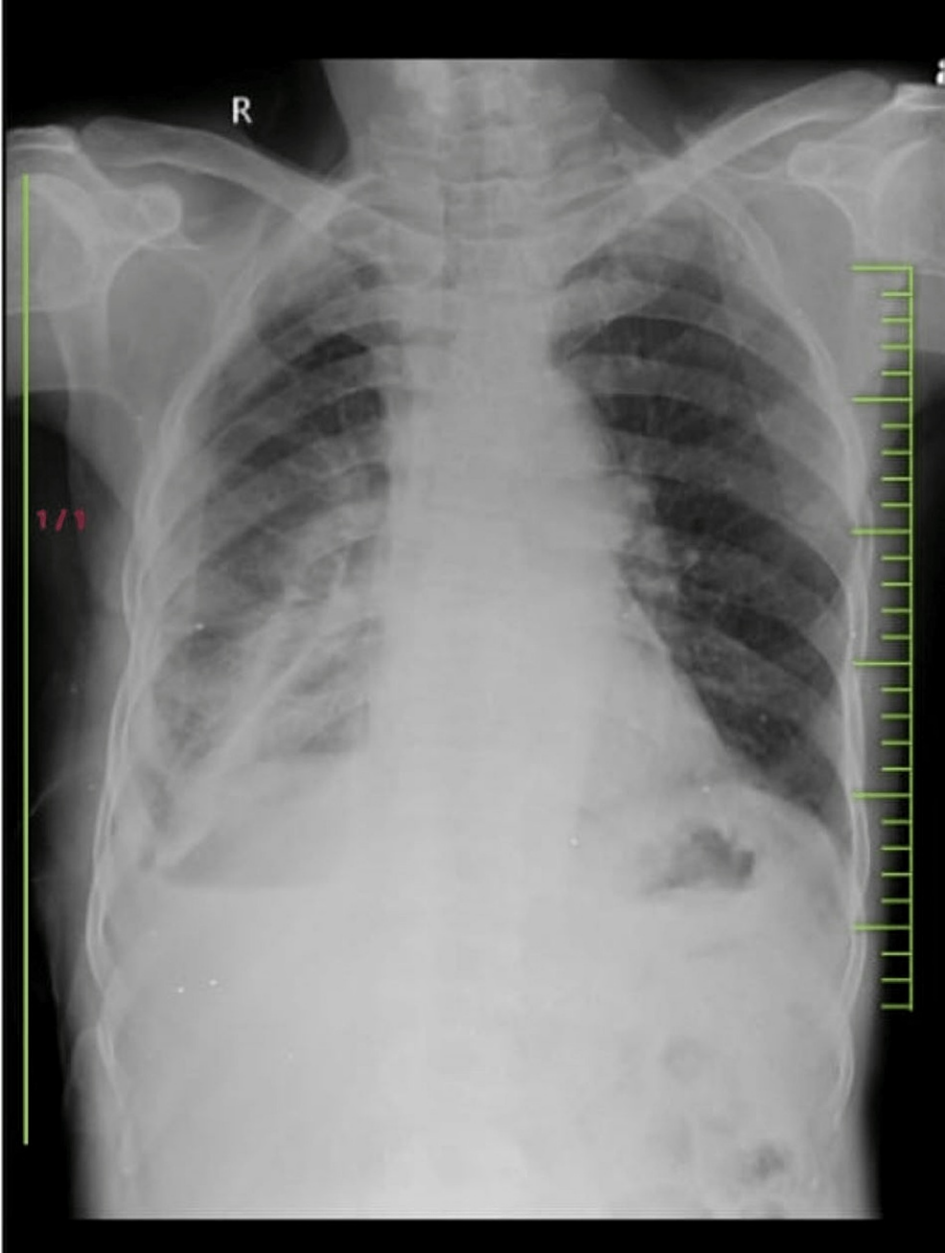
Chest X-ray of the patient (posteroanterior view). Heterogenous opacification can be noted in the right lower lobe of the lung with air bronchogram. Right lower lobe consolidation can be noted along with loculated right-sided pleural effusion.

A 72-year-old male, farmer by occupation, came to the hospital with complaints of dry cough, breathlessness on walking, right-sided chest pain and upper back pain for 15 days, and high-grade fever for 15 days. The pain progressed gradually. The patient also provided a history of low-grade fever for two days. After the development of these symptoms, he was taken to a private hospital near his residence where medications were prescribed. The patient was a chronic bidi smoker for 30 years, smoking three to four bidis per day. In the hospital, he underwent computed tomography (CT) scan of the thorax and was advised a thoracoscopy. During the procedure, 100 mL of thick pus was aspirated through thoracoscopy. Post-thoracoscopy, when the patient was stable, a clinical examination was done. He was in supine lying position, conscious, and well-oriented to time, place, and person with a mesomorphic build. During observation, a Foley catheter and an intercostal drainage tube (ICD) number 28 were present, and chest movements were decreased. On examination, pulse rate was 104 beats/minute, blood pressure was 130/88 mmHg, and oxygen saturation was 98%. His breathing pattern was abdomino-thoracic type with a respiratory rate of 23 breaths/minute. On palpation, the chest was asymmetrical, chest expansion was decreased, and the trachea had shifted to the left side. Stony dullnote and decreased vocal resonance over the right side were noted when percussed. The air entry was reduced on both sides, although more on the right. The patient was suspected for tubercular pleural empyema. Therefore, he underwent a high-resolution CT (HRCT) scan of the lungs on January 22, 2022. It revealed moderate right pleural effusion with loculations in places. Consolidation with sub-segmental compression atelectasis was seen in the underlying right lower and middle lobe region. Again, HRCT of the lung was done on January 27, 2022, which revealed, small air foci in the pleural collection, post-tapping status. Small patchy pneumonitis areas in both upper lobes were noted, suggestive of infectious etiology. The radiographical findings are shown in Figure 1. Later, 400 mL of pleural fluid was sent for cytopathological examination. It was reddish hemorrhagic fluid and suggested acute inflammatory leucocytic suppurative exudation like empyema. A high-dose contrast-enhanced computed tomography (CECT) scan of the thorax was done, which revealed a peripherally enhancing loculated large pleural collection noted on the right side causing the collapse of the right lung plus mediastinal shift and multiple enhancing lymph nodes, suggestive of empyema and right lung collapse. Other lab investigations were done; on complete blood count testing, the haemoglobin was reduced (10.9 g/dL). The kidney function test showed a reduced sodium level (128 mEq/L) and reduced creatinine level (0.6 mg/dL).

Therapeutic intervention

The objective of physiotherapy was to enable him to return to his everyday activities and health maintenance. The detailed physiotherapy rehabilitation protocol is presented in Table 1.

**Table 1:** Physiotherapy rehabilitation protocol.

Goals	Therapeutic interventions	Treatment protocol
Patient education	Educating the patient about exercises and its importance. Gaining cooperation and consent from the patient and his family	The patient and the caregiver were educated about the importance of positioning, ambulation, and functional activities of daily living
To improve bed mobility	Monitored for bed transitions and bedside sitting	Patient was taught rolling and bedside sitting. Positioning helped prevent bed sores, facilitate drainage, and improve ventilation which increased oxygen uptake
To retrain breathing pattern and reduce dyspnea	Controlled breathing exercises were taught, which included, pursed lip breathing and diaphragmatic breathing	The patient was advised to perform these exercises 10 times two to three times a day which improved the breathing efficiency
To improve lung volume	Thoracic expansion exercises, flexion of shoulder with deep inspiration, and expiration while extension	Ten repetitions in one set twice a day were prescribed
Active range of motion exercises for the upper and lower limbs	Range of motion exercises for all joints of the upper and lower limbs	Daily 8-10 repetitions for each joint actively. This maintained the joint mobility
To improve lung volume and capacity	Thoracic expansion exercises: shoulder in flexion with deep inspiration and extension with expiration. Incentive spirometer was used. Visual feedback through differently colored balls representing 600, 900, and 1200 cc	Initially 10 repetitions in one set twice a day; later, 10 repetitions in two sets three to four times a day. Initially, the patient was told to perform spirometry two to three times a day; later, the patient was suggested to perform spirometry every two hours
Early mobilization	Ambulation in the hallway	Early mobilization helps in improving the functional residual capacity

Follow-up and outcome measures

Table 2 presents the detailed follow-up and outcome measures of the patient.

**Table 2:** Follow-up and outcome measures.

Outcomes	First day of referral	At the time of discharge	Follow-up
Grades of dyspnea	II	I	I
St. George’s Respiratory Questionnaire	76	58	55
Hospital Anxiety and Depression Scale	10	6	5

## Discussion

Empyema is the collection of pus in the pleural cavity most commonly caused by pre-existing lung diseases such as bacterial pneumonia, tuberculosis, lung abscess, or bronchiectasis. Direct infection into the pleural space is the most common cause [5]. Clinical signs include continuing fever with signs of pleural involvement. The goals of the treatment are to eliminate the infection, obtain full lung expansion, and prevent the development of a rigid chest wall. Pleural aspiration, instillation of antibiotics, and physiotherapy are the daily treatment plan for patients [3]. Pleural effusion occurs when fluid settles in the pleural cavity. It can occur by transudation or exudation. It may be asymptomatic or associated with pleuritic pain [6]. Empyema can be a secondary cause of pleural effusion. When patients experience such conditions, a physiotherapist can assist in regaining mobility and function and improving their quality of life [7]. Chest physiotherapy has long been a standard aspect of treatment after a thoracoscopy [8] and includes patient education, early mobilization, splinted coughing or huffing, thoracic expansion exercises, using mechanical devices, and well-planned home exercise program during discharge [7,9]. We can conclude that flow-metric incentive spirometry, combined with breathing exercises and airway clearing techniques, can be effective in improving pulmonary function, forced vital capacity, and functional capacity. Chest physiotherapy has an essential role in both preventing and treating pulmonary problems.

## Conclusions

It has been demonstrated that pulmonary rehabilitation improves patient ventilation and reduces dyspnea. It helps in the faster recovery of patients. In this case, after the rehabilitation, the patient was able to perform functional activities independently without feeling exhausted. The breathlessness was also reduced post-rehabilitation. Even though complete recovery was not achieved following rehabilitation, the patient’s lung vital capacity and exercise tolerance were significantly improved.
